# A modified X10-23 DNAzyme that can better access large, structured RNA targets

**DOI:** 10.1093/nar/gkaf1449

**Published:** 2026-01-06

**Authors:** Connor Nurmi, Halle M Barber, Harneesh Kaur, John D Brennan, Masad J Damha, Yingfu Li

**Affiliations:** Department of Biochemistry and Biomedical Sciences, McMaster University, Ontario L8S 4L8, Canada; Biointerfaces Institute, McMaster University, Ontario L8S 4L8, Canada; Department of Chemistry, McGill University, Montreal, Quebec H3A 0G4, Canada; Department of Chemistry, McGill University, Montreal, Quebec H3A 0G4, Canada; Biointerfaces Institute, McMaster University, Ontario L8S 4L8, Canada; Department of Chemistry, McGill University, Montreal, Quebec H3A 0G4, Canada; Department of Biochemistry and Biomedical Sciences, McMaster University, Ontario L8S 4L8, Canada

## Abstract

The 10–23 DNA enzyme is one of the most efficient RNA-cleaving enzymes reported, possessing substrate recognition arms that can be designed to target virtually any AU diribonucleotide junction. However, 10–23 often shows reduced activity for large, structured RNA (lsRNA) substrates like messenger RNA. Increasing arm length or adding antisense DNA oligonucleotides (ASOs) can improve accessibility to lsRNA but may also reduce the efficiency of product release. Xeno nucleic acids (XNAs), such as 2′-fluoro-arabinonucleic acid (FANA), have been substituted for DNA into the arms of 10–23 to improve activity, such as in the FANA-modified X10-23, but X10-23 also shows poor accessibility for lsRNA targets. To overcome this issue, we substituted patterns of various XNAs with high RNA binding strength into the substrate recognition arms of X10-23. We found that an X10-23 enzyme with a distinct 2′F-RNA-LNA-FANA arm pattern, denoted as XdZ-2, could gain access to several lsRNA targets from SARS-CoV-2, achieving cleavage rates up to 82-fold faster than X10-23 for one system. While the ASO strategy provided higher cleavage rates for two other lsRNA systems, XdZ-2 may be a more attractive alternative in low Mg^2+^ environments and in terms of improving the efficiency of product release and stability in biological samples.

## Graphical Abstract

**Graphical Abstract:**
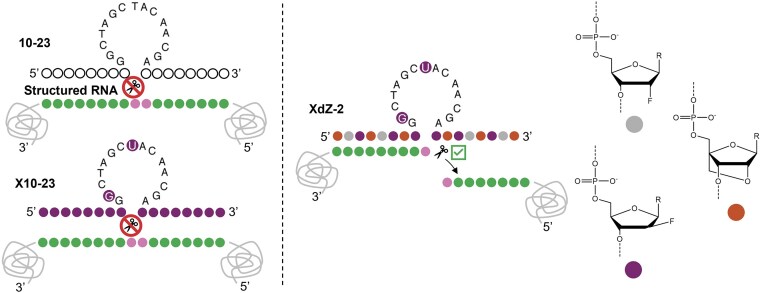

## Introduction

The ‘10–23’ DNA enzyme, also known as 10–23 DNAzyme, 10–23, or simply dZ, is a single-stranded DNA oligonucleotide that catalyzes the sequence-specific *trans* cleavage of RNA, employing modifiable substrate recognition arms that flank a central, 15-nt-long, catalytic core sequence [1]. 10–23 was developed by *in vitro* selection and remains one of the most efficient RNA-cleaving DNAzymes reported [2, 3]. However, the rate of RNA transesterification by 10–23 still falls behind protein enzymes and RNA-based catalysts (ribozymes), and as a result, many groups have explored various strategies to improve its activity.

Substitution of xeno nucleic acids (XNAs) into the substrate recognition arms and/or catalytic core of the 10–23 DNAzyme has been explored by other groups to improve RNA-cleavage activity [4, 5]. These studies primarily used XNAs with modifications to the 2′ position of the ribose sugar, demonstrating improved RNA heteroduplex stability compared to RNA:RNA and DNA:RNA duplexes. The first XNA to be substituted into the substrate recognition arms of 10–23 was 2′-OMe, which aimed to prevent exonucleolytic degradation in biological samples [6]. Since then, 2′-OMe [7, 8] and other XNAs, such as locked nucleic acids (LNA) [9–12] and 2′-fluoro-arabinonucleic acids (FANA) [4], have been explored for their ability to improve the RNA-cleavage activity of 10–23. The development of polymerase enzymes that are capable of accommodating XNA substrates has also given rise to fully XNA enzymes, including the all-arabinose (ANA) AR17_5, all-hexitol (HNA) HR16_1, all-cyclohexene (CeNA) CeR16_3, and all-FANA FR17_6 [13], FR6_1 [5], and NGS12-7 [14], though these span a large range of activities.

Although XNAzymes generally demonstrated improved RNA-cleavage activity compared to their DNAzyme counterparts, early XNAzymes, which possessed only 2′-OMe and LNA, displayed reduced cleavage activity with large, structured RNA (lsRNA) substrates compared to other XNAzymes containing FANA [4, 15]. Moreover, some XNA-based species, such as LNA oligomers, have been shown to elicit immunogenic responses or hepatotoxic effects *in vivo* [16]. As a result, many groups have focused on incorporating XNAs with 2′-fluorine modifications such as FANA and 2′F-RNA (FRNA) due to their lower immunogenicity and high RNA binding stability [17–19]. While the potential immunogenicity of these XNAzymes remains to be experimentally validated, they nonetheless provide an appealing substitute for DNA in the substrate recognition arms of the 10–23 DNAzyme.

There are two primary issues that prevent the widespread use of DNAzymes and XNAzymes for diagnostic and therapeutic applications: (1) reduced activity for lsRNA substrates, especially at lower concentrations of Mg^2+^ [20, 21], and (2) higher cost and laboriousness of synthesis of XNAzymes [22, 23]. XNAzymes incorporating FANA into the substrate recognition arms, such as the FANA-modified enzyme X10-23 and the all-FANA FR6_1 enzyme, exhibit high activity with short, unstructured RNA (sRNA) substrates, with catalytic rates (*k*_obs_) up to 0.68 min^−1^ for X10-23 and 0.75 min^−1^ for FR6_1 [4, 5], but are significantly less active with lsRNA substrates. Increasing the substrate recognition arm length is one potential solution to improve lsRNA accessibility. For example, to successfully target and cleave messenger RNA (mRNA) from *KRAS*, the 5′ and 3′ substrate recognition arms of FR6_1 were extended to 10-nt ([10 + 10]), with shorter arms showing significantly reduced activity.

Although longer arms increase lsRNA-cleavage activity through stronger substrate hybridization, they can be more laborious and expensive to synthesize, and show a reduced rate of product release (*k*_off_) [2, 24]—deleterious for downstream applications. Addition of “helper” antisense DNA oligonucleotides (ASOs) upstream and downstream of the lsRNA target site is another strategy to improve accessibility of lsRNA to 10–23 DNAzymes, as recently reported by our group [21]. While ASOs were effective at improving accessibility to lsRNA, the all-DNA system requires more nucleic acid species and may be more susceptible to degradation in biological samples or within cells compared to XNAs.

One strategy that may be a better alternative to the use of ASOs or longer substrate recognition arms is to substitute relatively short XNA sequences with diverse XNA patterns (XNA chimeras) that have high thermal stability with complementary RNA substrates into the arms of 10–23. In previous studies for therapeutic antisense and siRNA oligonucleotides, certain combinations of FRNA, FANA, and LNA significantly improved the hybridization strength to a complementary RNA target over all-DNA or all-FANA oligonucleotides, even at low Mg^2+^ concentrations (1–2 mM) [18, 25–27]. XNA chimeras are also a cheaper alternative compared to all-FANA XNAzymes due to the higher synthesis cost of FANA oligomers compared to oligomers of LNA and FRNA, with the latter being ∼23%–30% cheaper based on quotes from several custom oligonucleotide synthesis companies in the USA. Therefore, we hypothesize that highly stable RNA-hybridizing XNA chimeras, when incorporated into the substrate recognition arms of 10–23, may better access structured target sites within lsRNA substrates, despite their relatively short length.

To test this hypothesis, we used the FANA-modified enzyme X10-23, which has demonstrated high activity for sRNA substrates, but low lsRNA-cleavage activity, as a model system to better access lsRNA substrates [4]. We report an XNAzyme, XdZ-2, with a distinct pattern of LNA–FRNA–FANA that showed enhanced lsRNA-cleavage activity to several lsRNA targets compared to the original enzyme X10-23. XdZ-2 also demonstrated superior lsRNA cleavage activity compared to X10-23 under both high and low Mg^2+^ concentrations, showcasing its potential to access structured target sites within lsRNA for a variety of applications.

## Materials and methods

### Oligonucleotide synthesis

**Figure 1.**
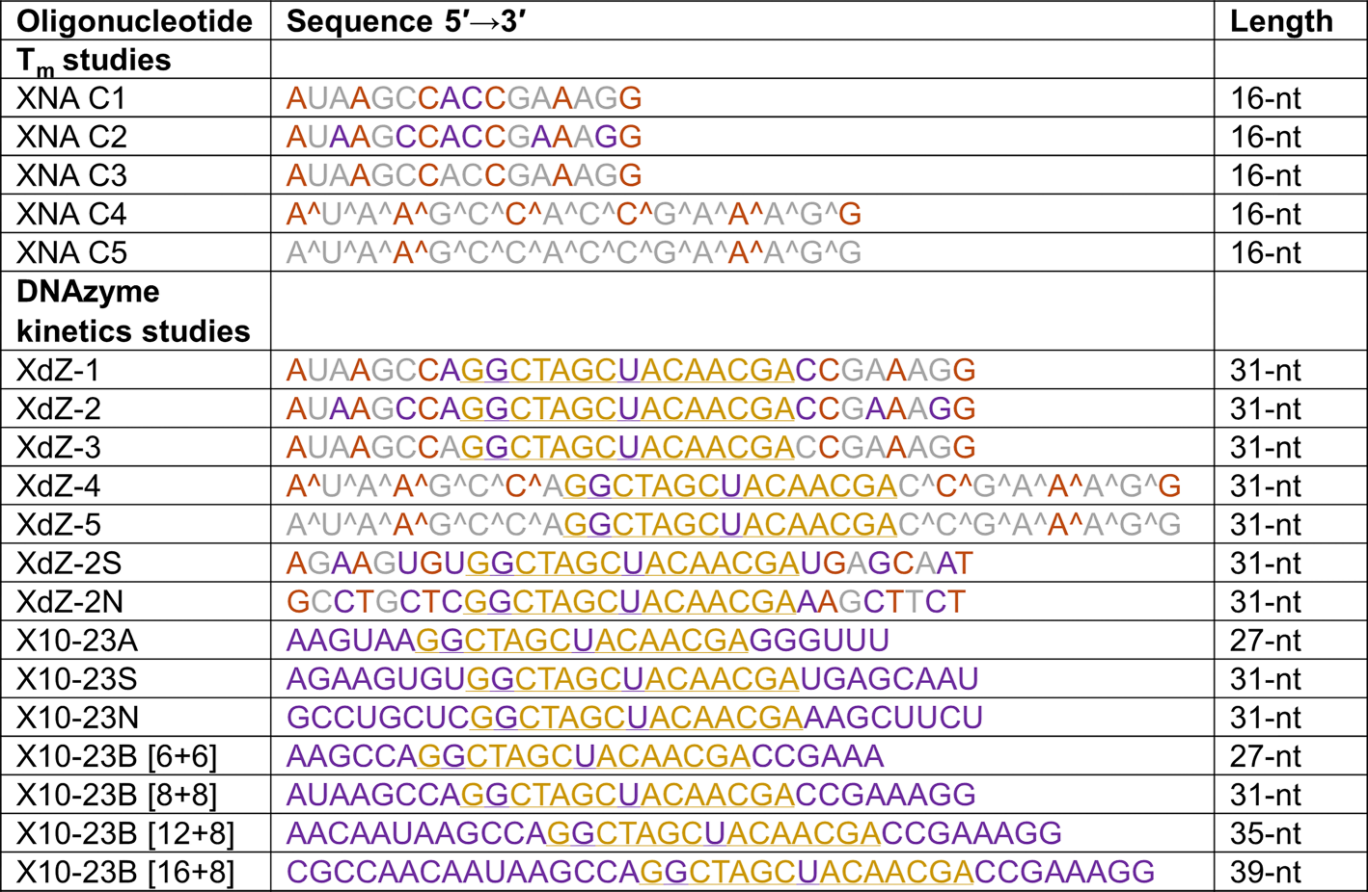
List of oligonucleotide sequences used in this study. Oligonucleotides were colored as follows: FANA (purple), FRNA (gray), LNA (orange), and DNA (yellow). All LNA cytidines are the 5-methylcytidine variant. Phosphorothioate linkages are denoted by the “^” symbol. Substrate recognition arm length is denoted within the brackets.

All unmodified DNA and RNA oligonucleotides were ordered from Integrated DNA Technologies (IDT) and purified by denaturing polyacrylamide gel electrophoresis (dPAGE), with sequences listed in Fig. 1 and Supplementary Table S1. Nucleic acids that contained modified nucleotides were synthesized on either an ABI 3400 DNA synthesizer (Applied Biosystems) or a MerMade 12 oligonucleotide synthesizer (LGC Biosearch Technologies) via solid-phase oligonucleotide synthesis. Phosphoramidites (ChemGenes) were prepared as 0.1 M solutions in acetonitrile (ACN), except for LNA 5-methyl cytidine, which was prepared in ACN:dichloromethane (DCM) (1:1, v/v). 500 Å Unylinker CPG was used as a solid support on a 1 µmol scale. A solution of 3% trichloroacetic acid in DCM was used to remove the dimethoxytrityl (DMTr) groups from the growing oligonucleotide strand at the beginning of each cycle and at the end of the synthesis. The phosphoramidites were activated for coupling with 0.25 M 5-ethylthio-1H-tetrazole in ACN. A coupling time of 3 min was used for all phosphoramidites except for FANA, for which 6 min was used. Sequences that failed to couple were capped with acetic anhydride in pyridine/tetrahydrofuran (THF) and 16% *N*-methylimidazole in THF. The sequences were either oxidized with 0.1 M iodine in pyridine/water/THF to create phosphodiester linkages or sulfurized with 0.1 M 3-[(dimethylamino-methylidene)amino]-3H-1,2,4-dithiazole-3-thione (DDTT) to create phosphorothioate linkages.

Following synthesis, the oligonucleotides were deprotected using ammonium hydroxide at room temperature for 48 h and filtered. The crude oligonucleotides were purified on a Waters 1525 anion-exchange HPLC using a Protein-Pak DEAE 5PW anion exchange column (21.5 mm × 150 mm) with a flow rate of 10 ml/min at 60°C using a gradient of 0%–100% buffer B (0.5 M LiClO_4_ and 10 mM NaOAc pH 5.5 in 25% ACN in water). The purified oligonucleotides were desalted using Gel Pak 2.5 size-exclusion columns (Glen Research), quantified using UV spectroscopy, and characterized using electrospray ionization LC-MS. Extinction coefficients were calculated using the IDT OligoAnalyzer tool.

### UV thermal melting studies

Ultraviolet (UV) thermal denaturation experiments were conducted on a Varian Cary 100 UV-visible spectrophotometer equipped with a Peltier temperature controller. The sample containing 2 nmols of each complementary oligonucleotide (2 µM duplex concentration) was annealed slowly in 5 mM sodium phosphate buffer containing 140 mM KCl and 1 mM MgCl_2_ (pH 7.5). The denaturation curves were acquired in triplicate in the temperature range of 10°C to 90°C using 260 nm wavelength at a ramp rate of 0.5°C per minute. The acquired spectral curves were normalized, and the melting temperature (T_m_) was calculated by taking the first derivative of the midpoint of the dissociation curves.

### Circular dichroism studies

Circular dichroism spectra were acquired on a Chirascan VX CD spectrophotometer (Applied Photophysics) using 1 mm path length cuvette at constant 21°C temperature. The oligonucleotides were dissolved in 5 mM sodium phosphate buffer solution containing 140 mM KCl and 1 mM MgCl_2_ (pH 7.4) and a final duplex concentration of 25 µM. Prior to analysis, the samples were annealed by heating at 90°C for 5 min and allowing them to cool overnight to room temperature. The spectral data were recorded in triplicate over a wavelength range of 200–320 nm, using parameters of 0.5 s time per point and 1 mm bandwidth. The average spectra were subtracted from blank buffer scan and smoothed using the Savitzky-Golay function within the Chirascan graphing software.

### RNA substrate preparation and γ-^32^P ATP radiolabeling

Short RNA substrates were ordered from IDT and prepared according to manufacturer instructions. The lsRNA substrates were generated from their corresponding DNA templates, which were purified using a New England Biolabs Monarch PCR and DNA clean-up kit, following amplification by polymerase chain reaction (PCR). The concentration of the PCR product was determined using a NanoVue Plus spectrophotometer by measuring A_260_ after the purity was verified by dPAGE. The lsRNA for each PCR-produced DNA product was *in vitro* transcribed using following reaction conditions at 37°C for 2 h: 2 pmol DNA template, 100 U T7 RNA polymerase, 5 mM DTT, 1× T7 RNA transcription buffer (20 mM Tris–HCl pH 7.9, 3 mM MgCl_2_, 5 mM DTT, 5 mM NaCl, and 1 mM spermidine), 0.2 units of pyrophosphatase, 2 mM nucleotide triphosphate (NTP) mixture, and 40 U of RiboLock (all from Thermo Scientific). The reaction was incubated for another 20 min at 37°C following the addition of 5 U of DNase I. The *in vitro* transcription products were then purified by dPAGE containing 8 M urea, imaged by UV shadow, and excised. The excised gel fragment was then soaked in a 1× elution buffer (200 mM NaCl, 10 mM Tris–HCl pH 7.5 and 1 mM ethylenediaminetetraacetic acid (EDTA) pH 8.0) after crushing and shaking at room temperature for 20 min, followed by purification by EtOH precipitation. The lsRNA pellet was then resuspended in nuclease-free H_2_O and quantified spectrophotometrically.

All RNA substrates were radiolabeled with ^32^P (PerkinElmer) on the 5′-terminus. For lsRNA substrates, dephosphorylation was performed with 2 pmol RNA added to 1 U of alkaline phosphatase (FastAP, ThermoFisher Scientific) in 10 µl of 1× FastAP buffer (10 mM Tris–HCl pH 8.0, 5 mM MgCl_2_, 500 mM KCl, 0.02% Triton X-100, and 0.1 mg/ml BSA; ThermoFisher Scientific) and incubated at 37°C for 30 min. The dephosphorylated lsRNA was processed by the addition of 5 µl of 3 M NaOAc and an equal volume of phenol-chloroform-isoamyl alcohol 25:24:1 pH 6.7/8.0 followed by shaking for 2 min. After centrifugation for 2 min, the upper aqueous layer was removed and washed twice with 50% chloroform. The resulting aqueous layer was then purified by EtOH precipitation and resuspended in a solution containing ∼10 µCi γ-^32^P ATP and 10 units of polynucleotide kinase (PNK, ThermoFisher Scientific) in 10 µl of 1× PNK buffer A (50 mM imidazole-HCl pH 6.4, 18 mM MgCl_2_, 5 mM DTT, 0.1 mM spermidine, and 0.1 mM ADP; ThermoFisher Scientific) and incubated at 37°C for 20 min. The reaction was purified by dPAGE containing 8 M urea and the gel was exposed on an Amersham Biosciences storage phosphor screen for 15 min. The screen was then imaged on an Amersham Typhoon 9200 scanner with a photomultiplier tube sensitivity of 4000 V, and the 5′-radiolabeled ^32^P RNA was excised, crushed, soaked in 1× elution buffer, and EtOH precipitated.

### DNAzyme kinetics reactions

All kinetic reactions were performed in duplicate at room temperature (23°C) under single-turnover conditions unless otherwise stated. Individual timepoints were taken from a master reaction mixture containing ∼1 pM of ^32^P-labeled RNA substrate and 500 pM DNAzyme in 10 µl of 1× buffer 1 (50 mM 4-(2-hydroxyethyl)-1-piperazineethanesulfonic acid (HEPES) pH 7.4, 10 mM MgCl_2_, and 100 mM NaCl) and quenched with 10 µl of 2× quenching buffer (10% sucrose, 0.5× TBE, 30 mM EDTA, 8 M urea, and 0.14 mg/ml bromophenol blue and xylene cyanol) to stop the reaction on ice. Before addition of 1× buffer 1, the DNAzyme annealed to the RNA substrate by heating for 1 min at 90°C and cooling to room temperature for 5 min, and a *t* = 0 timepoint was taken. Each kinetic reaction was analyzed by dPAGE with 8 M urea and the RNA cleavage product bands were quantified using ImageJ software. The fraction of RNA substrate cleaved was measured using the ratio of intensity between cleaved and total (uncleaved + cleaved) RNA for each reaction. The data were then fit to the one-phase exponential association equation, $Y = {Y}_{max}( {1 - {e}^{ - {k}_{obs}t}} )$, using GraphPad Prism 8 to obtain the first-order rate constant, *k*_obs_.

## Results and discussion

### X10-23 activity with lsRNA targets

**Figure 2.**
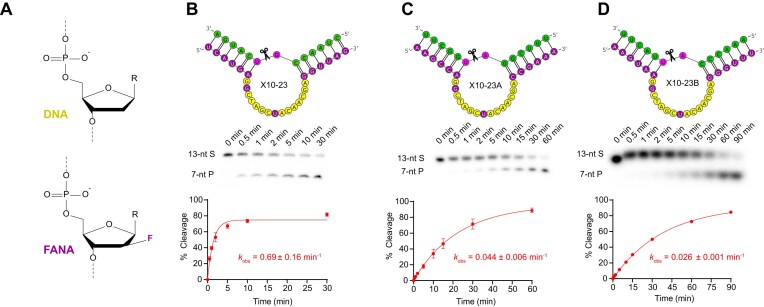
Structure comparison of DNA and FANA (**A**) used in the original “X10-23” DNAzyme developed by Wang and colleagues [4] (**B**). The RNA cleavage activity of X10-23 was compared to two variants that targeted different sites within the *ORF3a* gene of SARS-CoV-2, X10-23A (**C**) and X10-23B (**D**), with a complementary short, unstructured 13-nt RNA (sRNA) target. The X10-23 DNAzyme target site is shown in green, with A-U (diribonucleotides at cleavage site) shown in pink. The mainly DNA catalytic core of X10-23 is shown in yellow and FANA substrate recognition arms are shown in purple. Reactions were conducted at room temperature in buffer containing 10 mM Mg^2+^ and resolved in 10% dPAGE with 8 M urea.

Enzyme X10-23 is a FANA-modified version of the all-DNA 10–23 DNAzyme, with FANA substrate recognition arms and FANA at two positions (G_2_ and T_8_) within the catalytic core of 10–23 [4] (Figs 1 and 2A and B). Through a rational design approach, FANA substitutions at G_2_ and T_8_ improved RNA-cleavage activity and were thus implemented into X10-23. FANA was chosen because it adopts a DNA-like (C2′/O4′-*endo*) conformation while exhibiting high binding affinity toward both RNA and DNA targets as previously mentioned [28]. X10-23 showed higher *in vitro* RNA-cleavage activity compared to all-DNA 10–23, cleaving a short, unstructured 13-nt RNA target (sRNA) from *KRAS* mRNA with a *k*_obs_ of 0.69 min^−1^ (Fig. 2B).

To test the generalizability of X10-23 with sRNA, we designed two X10-23 variants, X10-23A and B, possessing the same 5′ and 3′ FANA substrate recognition arm length of 6 nucleotides [6 + 6], but targeting different sRNAs (13-nt) from the *ORF3a* gene of SARS-CoV-2. We observed a 16-fold and 26-fold decrease in sRNA-cleavage activity, respectively, for X10-23A (*k*_obs_ of 0.044 min^−1^) and X10-23B (*k*_obs_ of 0.026 min^−1^) compared to the original X10-23 (Fig. 2C and D). While all three X10-23 constructs were active with sRNA, there was some variation in cleavage activity, which is likely a result of intramolecular folding of X10-23, slowing the rate of association (*k*_on_) [20].

**Figure 3.**
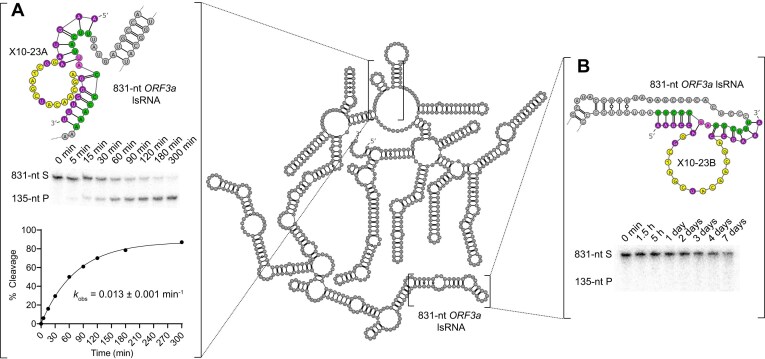
RNA cleavage activity comparison of two X10-23 variants, X10-23A and X10-23B, with a large, structured RNA (lsRNA) substrate from the *ORF3a* gene of SARS-CoV-2. Both X10-23A and B possessed 5′ and 3′ substrate recognition arm lengths of 6-nt [6 + 6]. X10-23A cleaved a relatively accessible target site within lsRNA with a *k*_obs_ of 0.013 min^−1^ (**A**), while X10-23B was completely inactive after 7 days (**B**). The target sites are shown in green, with A-U (diribonucleotides at cleavage site) shown in pink. The mainly DNA catalytic core of X10-23 is shown in yellow and FANA substrate recognition arms are shown in purple. Reactions were conducted at room temperature in buffer containing 10 mM Mg^2+^ and resolved in 10% dPAGE with 8 M urea.

Compared to sRNA, the catalytic performance of X10-23 with lsRNA (∼2100-nt *KRAS* mRNA) has been shown to be relatively poor based on follow-up studies [4, 29]. As such, there is some debate on the exact mechanism of gene silencing by X10-23, based on the fact that RNase H-mediated degradation of the ∼2100-nt *KRAS* mRNA transcript still occurred with an inactivated X10-23 negative control (we encourage the reader to refer to these follow-up studies and correspondence for more insight) [29, 30]. Nevertheless, to determine if X10-23 was generalizable to other lsRNA targets, both X10-23A and X10-23B were used to target different sites within the same 831-nt lsRNA transcript from the *ORF3a* gene of SARS-CoV-2. X10-23A targeted a region of the lsRNA transcript that was found to be more accessible than the target site of X10-23B, based on kinetic and structural data from previous studies in our lab [20, 21]. With X10-23A, we observed a three-fold decrease in activity for the 831-nt lsRNA substrate compared to the 13-nt sRNA substrate (Fig. 3A), while no lsRNA-cleavage activity was observed for X10-23B, even after 7 days (Fig. 3B), validating our hypothesis that lsRNA accessibility has a significant impact on X10-23 activity.

### Optimal X10-23 substrate recognition arm length

Increasing the X10-23 FANA substrate recognition arm length may improve accessibility to lsRNA because of increased thermodynamic stability of RNA hybridization [31], but longer arms are typically more expensive to synthesize and may reduce the release rate (*k*_off_) of the cleaved RNA products [2, 24], limiting the efficiency of downstream reactions. Reduced *k*_off_ can impact the overall performance of DNAzymes and XNAzymes under multi- and single-turnover conditions, but for most applications, only single-turnovers are necessary as the catalyst can be provided in excess of substrate. In some cases, 10–23 DNAzymes with relatively long DNA substrate recognition arms can still show low lsRNA-cleavage activity as a result of extensive intramolecular folding [2]. Using a variant of X10-23 as an example, the full-DNA version of X10-23B with [16 + 8] substrate recognition arms showed a *k*_obs_ of only 0.00017 min^−1^ after 10 days, with further reductions in arm length resulting in completely inactive DNAzymes (Supplementary Fig. S1). Taken together, we investigated the optimal FANA substrate recognition arm length with X10-23B to determine which size should be used for the XNA chimeric substitutions, ideally striking a balance between length (high *k*_off_, cheaper) and lsRNA cleavage activity (high *k*_obs_).

**Figure 4.**
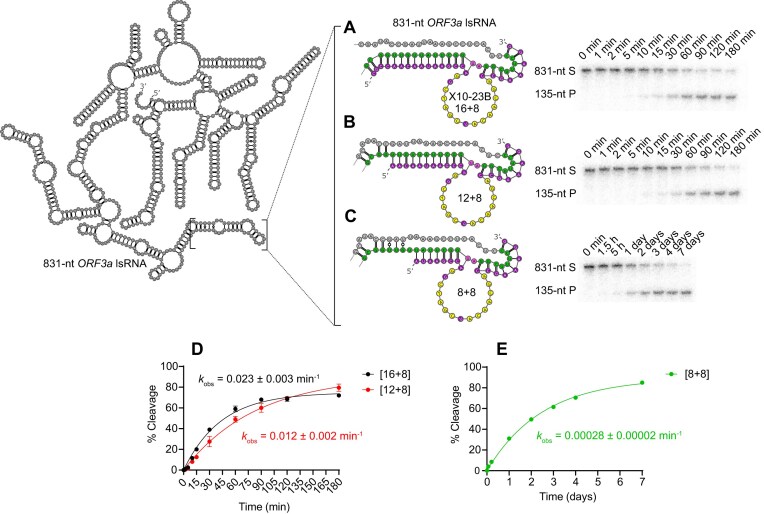
RNA cleavage activity of the X10-23 variant, X10-23B, with increasing FANA substrate recognition arm length for an 831-nt lsRNA substrate from the *OFR3a* gene of SARS-CoV-2. The rate of lsRNA cleavage for three X10-23 enzymes was tested with variable 5′ and 3′ FANA substrate recognition arm lengths. X10-23B with 5′ and 3′ lengths of [16 + 8] (**A**), [12 + 8] (**B**), and [8 + 8] (**C**) were tested, with [16 + 8] and [12 + 8] possessing relatively high lsRNA-cleavage activity (**D**), while [8 + 8] showed significantly lower lsRNA-cleavage activity (**E**). The X10-23 target site is shown in green with A-U (diribonucleotides at cleavage site) shown in pink. The mainly DNA catalytic core of X10-23B is shown in yellow and FANA substrate recognition arms are shown in purple. Reactions were conducted at room temperature in buffer containing 10 mM Mg^2+^ and resolved in 10% dPAGE with 8 M urea.

To find the optimal substrate recognition arm length, we designed three different X10-23B constructs with FANA [8 + 8], [12 + 8], and [16 + 8] arms, targeting the same 831-nt lsRNA substrate from the *ORF3a* gene of SARS-CoV-2. The 3′ arms were limited to 8-nt because a longer substrate recognition arm may decrease the *k*_off_ for downstream applications [2, 24]. Additionally, it has been shown that 10–23 DNAzymes with longer, asymmetric substrate recognition arms, are slightly more active compared to the same-size symmetric binding arms [31]. We observed higher lsRNA cleavage activity by X10-23B with FANA [16 + 8] substrate recognition arms (Fig. 4A) compared to FANA [12 + 8] (Fig. 4B) and FANA [8 + 8] (Fig. 4C). Kinetic analysis showed that X10-23B with FANA [16 + 8] cleaved lsRNA with a 2-fold faster rate than FANA [12 + 8] (*k*_obs_ = 0.023 min^−1^ vs. *k*_obs_ = 0.012 min^−1^) (Fig. 4D). A more significant 43-fold decrease in lsRNA cleavage activity was observed between FANA [12 + 8] and FANA [8 + 8] (*k*_obs_ = 0.012 min^−1^ versus *k*_obs_ = 0.00028 min^−1^), with FANA [8 + 8] appearing to be the lowest limit of X10-23B substrate recognition arm size before complete loss of lsRNA cleavage activity (Fig. 4E). Based on this data, we chose [8 + 8] as a model substrate recognition arm size for optimization experiments with XNA chimeras, striking a balance between retaining some lsRNA cleavage activity with the benefits of shorter substrate recognition arms. Our decision to not use arm lengths below [8 + 8] was also based on findings from previous XNA studies, which showed that FANA substrate recognition arms below 8-nt were unable to access target sites within lsRNA substrates [5].

### RNA-binding strength of chimeric XNA sequences

**Figure 5.**
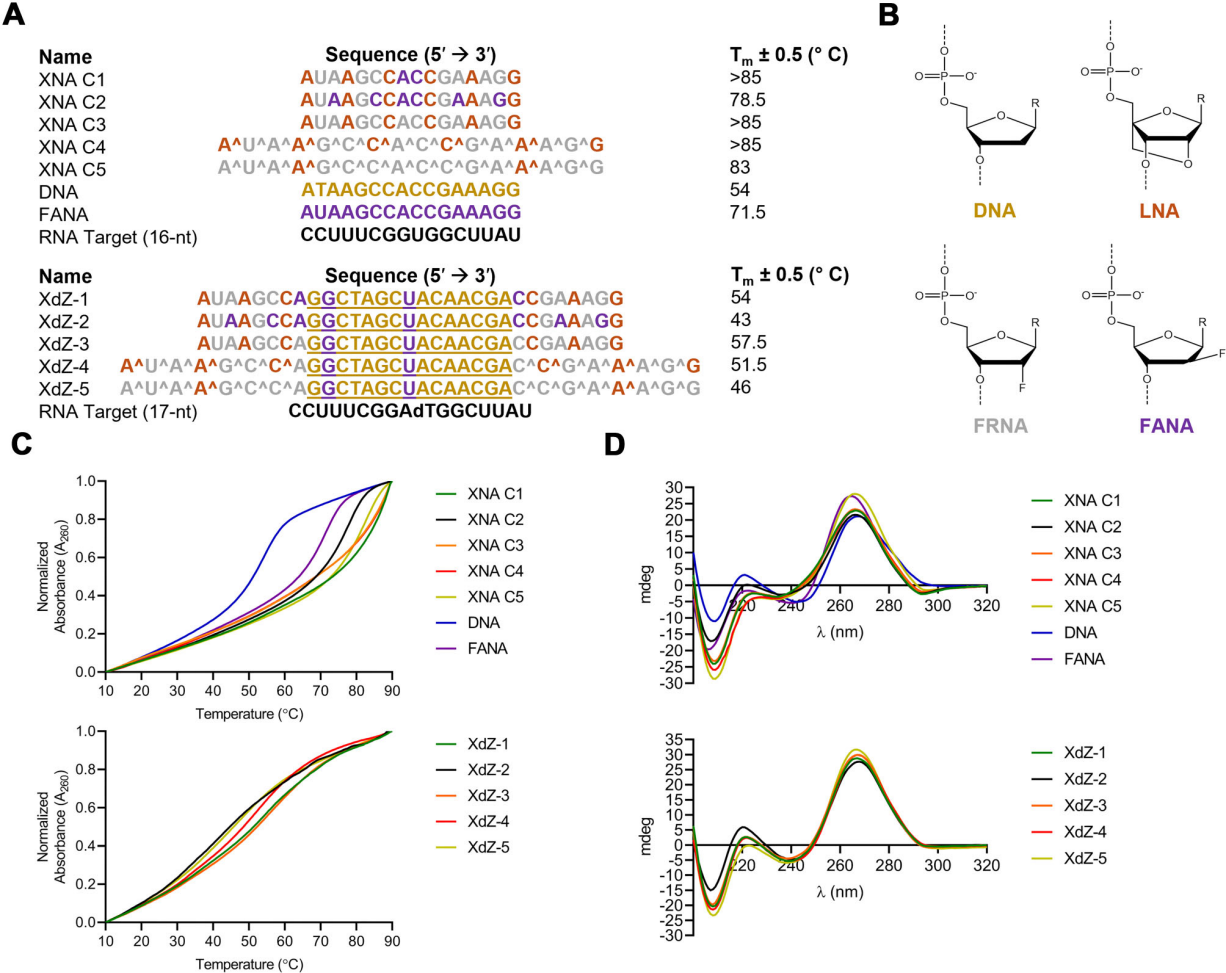
XNA and complementary RNA target sequences that were used in this study with the corresponding melting temperature (T_m_), with the oligomer (upper) and XNAzyme (lower) (**A**). The nucleic acid colors correspond to the following XNA structures in panel (**B**). UV thermal stability plot for each sequence in panel (A) targeting the same complementary RNA sequence (**C**) with corresponding circular dichroism spectra (**D**). The 10–23 DNAzyme catalytic core is underlined and XNAs are colored as follows: DNA (yellow), FANA (purple), LNA (orange), and FRNA (gray). Phosphorothioate linkages are indicated by “^.”

To improve lsRNA accessibility to X10-23 using a substrate recognition arm optimization strategy, we turned to previous studies that have shown that certain oligonucleotides with unique patterns of XNAs (XNA chimeras) possess stronger hybridization (higher T_m_) to RNA targets compared to all-DNA or all-FANA oligonucleotides [18, 25–27]. From these data, we hypothesized that incorporating XNA chimeric sequences with high T_m_ values into the substrate recognition arms of [8 + 8] X10-23B will improve lsRNA-cleavage activity more than the original X10-23 with longer all-DNA or all-FANA substrate recognition arms. While we postulate that the XNA chimeric sequences will provide simultaneous benefits of strong association to the lsRNA target (*k*_on_) and rapid product release (*k*_off_) following cleavage, the exact parameters will require experimental validation in more detailed kinetic studies. We thus designed five unique XNA chimeric sequences (Fig. 5A) and measured their hybridization strength with an sRNA target. All five of the XNA chimeras contained unique combinations of FANA, FRNA, and LNA, with two of these containing additional phosphorothioate linkages [32, 33], which have been shown to improve nuclease resistance within biological samples or *in vivo* (Fig. 5B). One example is the FANA–LNA–FRNA pattern in XNA C1, which was adopted from the therapeutic antisense XNA oligonucleotide (X-ASO), PO-G2B, possessing a ΔT_m_ 8.3°C higher with a complementary RNA target compared to the same all-FANA version [27]. Another X-ASO, PO-LNAFRNA, which consisted of an FRNA–FRNA–LNA pattern used in XNA C3 and C4, had a ΔT_m_ over 16°C higher with a complementary RNA target compared to the same all-FANA version.

We first performed UV thermal stability experiments on the five distinct XNA chimeras with a complementary sRNA target, both with (31-mer) and without (16-mer) the X10-23 DNAzyme catalytic core sequence (Fig. 5C). For the 16-mer XNA sequences, we observed that four of the five XNA chimeras (XNA C1 and C3-5) possessed relatively high T_m_ values >80°C with the sRNA target. We hypothesized that high relative abundance of LNA and FRNA was the main cause of the high sRNA binding strength for these XNA chimeras, which form the strongest interactions with RNA (ΔT_m_ + 3 to + 10°C/LNA modification, ΔT_m_ + 2 to + 3°C/FRNA modification) compared to FANA (ΔT_m_ + 0.5 to + 2°C/FANA modification) [34–36]. Mechanistically, the increase in the RNA hybridization strength by LNA and FRNA is due to their adoption of the same C3′-*endo* “*North*” sugar pucker as RNA, matching the same A-form structure of target RNA. The replacement of the hydroxyl group with fluorine in FRNA further increases the strength of RNA hybridization through 2′-hydrogen polarization [37]. The same trend was observed for the 31-mers, except for the low T_m_ observed for XdZ-5, which may have been caused by destabilization effects from the X10-23 catalytic core sequence.

XNA C2, which possessed an alternating LNA–FRNA–FANA pattern, had the lowest T_m_ of all the XNA 16-mers, at 78.5°C, but was still higher than the all-DNA (54°C) and FANA (71.5°C) 16-mers. The same trend was seen for the corresponding 31-mer, XdZ-2, which also had the lowest T_m_ of all the XdZ 31-mers. The decrease in RNA hybridization strength for XNA C2 and XdZ-2 compared to the other XNA chimeras may be a result of several adjacent FANA and FRNA moieties, which form A/B junctions that arise from competing “*North*” and “*South*” sugar pucker conformations, which can have destabilizing effects on duplex formation [18, 38, 39]. Adjacent LNA and FANA moieties have also been shown to form A/B junctions, further decreasing RNA hybridization stability [40–42], although it is also possible that LNA may influence FANA to adopt a more “*North*” sugar pucker conformation, as was observed in adjacent LNA–DNA moieties [43]. Regardless, A/B junctions may explain the relatively lower T_m_ values of XNA C2:RNA and XdZ-2:RNA duplexes.

We also aimed to evaluate whether phosphorothioate (PS) linkages could improve RNA-binding strength compared to canonical phosphodiester linkages. It has been shown that PS oligonucleotides generally form less stable complexes with RNA, but hybridization strength can be improved with the addition of XNAs with 2′-modifications like LNA, FRNA, and FANA [44, 45]. We developed two XNA chimeras with PS linkages, 16-mer XNA (C4 and C5) and their 31-mer counterparts (XdZ-4 and XdZ-5), and tested their hybridization stability to the complementary sRNA target. Both XNA C4 and C5 showed high T_m_ values >80°C. By contrast, the 31-mers XdZ-4 and XdZ-5 showed decreased RNA binding strength compared to the other 31-mers, except for XdZ-2, indicating that PS linkages will likely not offer much benefit to improving X10-23 generalizability and accessibility to structured target sites.

Finally, circular dichroism (CD) spectra were acquired to gain more insight into the helical conformation of each 16-mer XNA chimera and 31-mer XdZ sequence. The DNA B-form helix typically exhibits a broad positive band between 260 and 280 nm with a negative band at 245 nm, while the RNA A-form helix has a positive band at 270 nm and a negative band at 210 nm [46]. Hybrids of DNA:RNA exhibit more A-like helical structure with a broader positive 270 nm band and smaller negative 210 nm band [47]. Unsurprisingly, all XNA C1-C5 and XdZ-1–5 molecules displayed characteristics of an A-like helix, characterized by prominent negative bands at 210 nm (Fig. 5D). Since differences in RNA hybridization strength occurred between XNA C2 and XdZ-2 compared to the other XNA chimeric molecules, without major differences in overall helical structure from CD spectra analysis, there likely exist small conformational changes that play subtle, yet important roles in the strength of RNA binding.

### RNA-cleavage activity of chimeric X10-23 enzyme sequences

Following the UV-thermal stability and CD experiments, we performed single-timepoint kinetics with each XdZ chimera (XdZ-1 to 5) to evaluate the relationship between RNA hybridization strength and RNA-cleavage activity, using 17-nt sRNA and 831-nt lsRNA transcripts from the *ORF3a* gene of SARS-CoV-2 as an initial system. The original X10-23 enzyme with FANA [8 + 8] substrate recognition arms (X10-23B) was also tested as a positive control.

**Figure 6.**
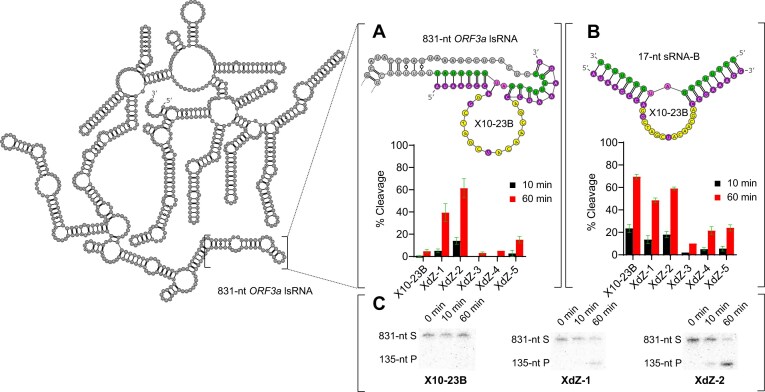
RNA cleavage activity comparison of the chimeric XNAzymes (XdZ) with [8 + 8] substrate recognition arms compared to X10-23B. Each XNAzyme was tested against an 831-nt lsRNA substrate (**A**) and short, 17-nt RNA substrate (sRNA-B) (**B**) from the *ORF3a* gene of SARS-CoV-2. Two chimeric XNAzymes, XdZ-1 and XdZ-2, showed higher lsRNA-cleavage activity compared to X10-23B (**C**). The chimeric XNAzymes possessed the same XNA sequence patterns from the UV thermal melt studies and were incorporated into the substrate recognition arms of X10-23. A-U (diribonucleotides at cleavage site) are shown in pink and the mainly DNA catalytic core of X10-23 is shown in yellow. Reactions were conducted at room temperature in buffer containing 10 mM Mg^2+^ and resolved in 10% dPAGE with 8 M urea.

We found that XdZ-1 and XdZ-2, reflecting the same substrate recognition arm patterns of XNA C1 and C2, respectively, cleaved 39% of the 831-nt lsRNA substrate (Y_60_ = 39%) and 61% (Y_60_ = 61%) after 60 min, respectively, based on the initial activity screen (Fig. 6A). X10-23B cleaved only 4% of lsRNA after 60 min (Y_60_ = 4%), indicating that XdZ-1 and XdZ-2 were much more effective at gaining accessibility to the lsRNA target site than with the original all-FANA arms. XdZ-3 and XdZ-4, which shared the same XNA chimeric sequence with either a PO (XdZ-3) or PS (XdZ-4) backbone, had similar activity to X10-23B. Lastly, XdZ-5, which was an entirely all-FRNA sequence with a central LNA nucleotide and PS backbone, was able to access the lsRNA substrate more efficiently compared to X10-23B, XdZ-3, and XdZ-4, with 15% cleaved after 60 min (Y_60_ = 15%).

Against the sRNA substrate (sRNA-B), X10-23B was the most active out of all the chimeric XNAzyme variants tested, cleaving 68% of sRNA-B after 60 min (Y_60_ = 68%) (Fig. 6B). The difference in X10-23B activity between the sRNA and lsRNA substrates highlights a significant drawback of using all-FANA oligonucleotides to target structured target sites compared to the chimeric XNA patterns of XdZ-1 and XdZ-2. FANA alone can easily hybridize to single-stranded RNA but appears unable to overcome the thermodynamic stability of an RNA–RNA duplex within lsRNA. However, when used in combination with LNA and FRNA nucleotides, as in XdZ-1 and XdZ-2, the chimeric XNA strand can overcome the energetic barrier and form more favorable interactions with lsRNA (Fig. 6C). This was further verified with native hybridization experiments comparing the extent of XdZ-2 and X10-23B hybridization to the lsRNA target, showing that a higher fraction of XdZ-2 was bound to lsRNA compared to X10-23B (Supplementary Fig. S2). Conversely, XdZ-3, XdZ-4, and XdZ-5 showed relatively poor activity with both sRNA and lsRNA substrates, indicating their respective substrate recognition arm XNA chimeric patterns poorly hybridized to structured or unstructured targets, possibly due to extensive intramolecular folding into inactive XNAzyme conformations.

**Figure 7.**
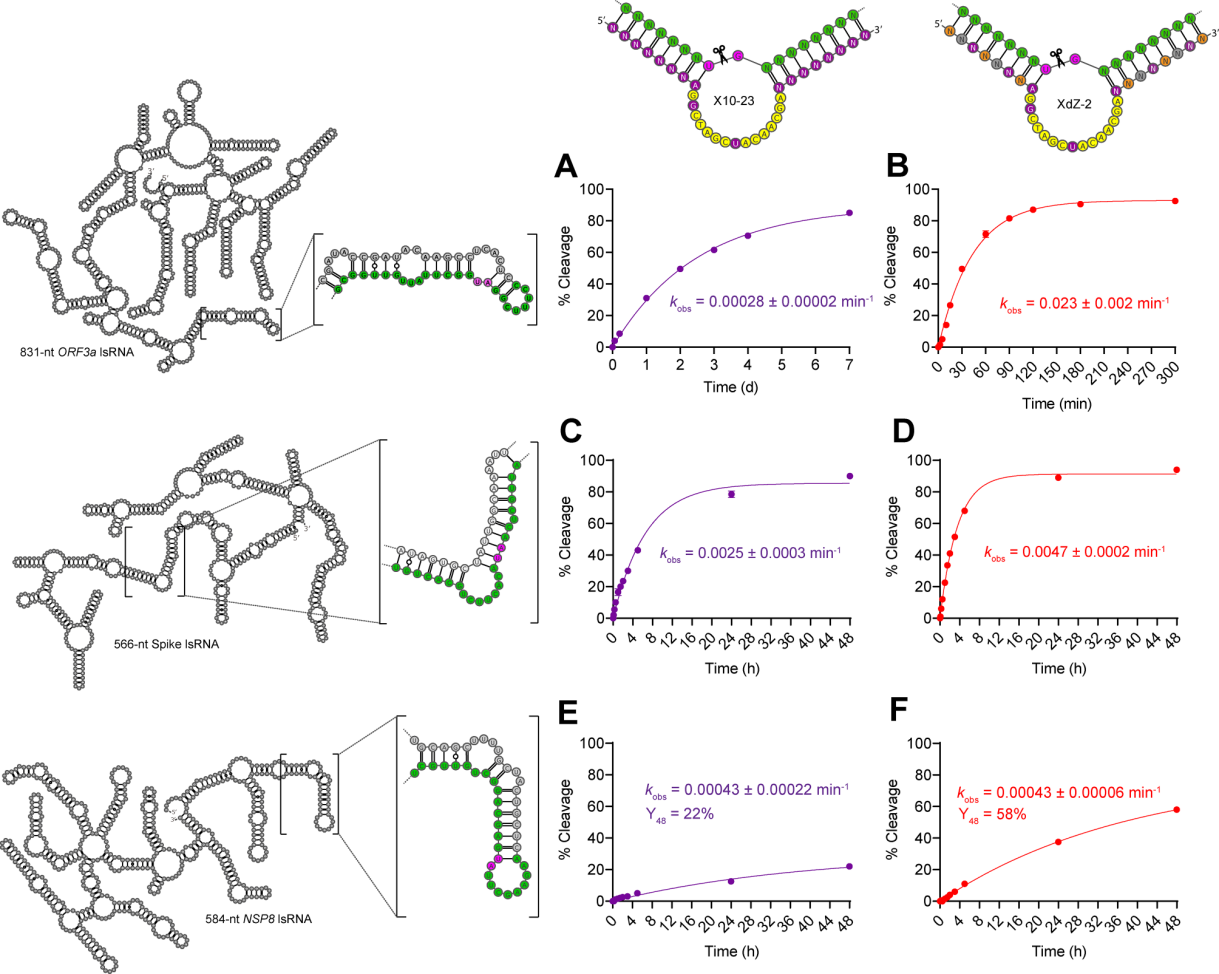
Activity comparison between X10-23 and XdZ-2 targeting three different lsRNA transcripts from the SARS-CoV-2 genome. X10-23B cleaved an 831-nt *ORF3a* lsRNA transcript with a *k*_obs_ of 0.00028 min^−1^ (**A**), while XdZ-2 cleaved the same target 82-fold faster with a *k*_obs_ of 0.023 min^−1^ (**B**). X10-23S cleaved a 566-nt Spike lsRNA transcript with a *k*_obs_ of 0.0025 min^−1^ (**C**), while XdZ-2S cleaved the same target 1.9-fold faster with a *k*_obs_ of 0.0047 min^−1^ (**D**). Both X10-23N (**E**) and XdZ-2N (**F**) cleaved a 584-nt *NSP8* lsRNA transcript with the same *k*_obs_ of 0.00043 min^−1^. All reactions were conducted at room temperature in buffer containing 10 mM Mg^2+^.

Since XdZ-1 and XdZ-2 possessed the highest fraction of lsRNA cleaved after 60 min (Y_60_) out of all other XNAzymes tested, they were chosen for more detailed kinetic analysis. With the 831-nt lsRNA substrate, XdZ-2 achieved a *k*_obs_ of 0.023 min^−1^, while XdZ-1 cleaved the same lsRNA substrate with a ∼2-fold slower rate with a *k*_obs_ of 0.014 min^−1^ (Supplementary Fig. S3). Compared to the rate of lsRNA cleavage by X10-23B (*k*_obs_ = 0.00028 min^−1^) (Fig. 7A), XdZ-2 cleaved the same lsRNA target 82-fold faster (Fig. 7B), highlighting the improved RNA duplex strand invasion properties of these XNA chimera arms. Between XdZ-1 and XdZ-2, significant differences in the maximum fraction of lsRNA cleaved (*Y*_max_) were observed, with *Y*_max_ = 70% for XdZ-1 and *Y*_max _= 93% for XdZ-2. We hypothesized that the difference in *Y*_max_ was a result of more extensive misfolding with XdZ-1 compared to XdZ-2. To confirm this, we performed native folding of each XNAzyme chimera and observed qualitative differences between XdZ-1 and XdZ-2 using native PAGE (Supplementary Fig. S4). The larger smearing pattern observed with XdZ-1 may be indicative of greater conformational heterogeneity compared to XdZ-2; however, the elucidation of the exact mechanism underlying these observations will require more in-depth structural studies.

While the results of the kinetics studies clearly show that the unique LNA–FRNA–FANA substrate recognition arm patterns of XdZ-1 and -2 are superior to XdZ-3, -4, and -5 at gaining access to lsRNA, the exact mechanism is unclear. The A/B junctions that arise from competing “*North*” and “*South*” sugar pucker conformations of FANA and FRNA, may provide unique RNA strand invasion properties, but additional physiochemical studies will be required to confirm the exact mechanism. We hypothesized that XNA chimeras with high RNA binding strength (high T_m_) would be able to overcome the thermodynamic stability of the RNA:RNA duplex of the target site within lsRNA, but unexpectedly, we observed the opposite phenomenon, where the lowest T_m_ XNAzymes (XdZ-1 and -2) had the highest lsRNA cleavage activity. In previous studies that used similar XNA patterns as X-ASOs to reduce gene expression [18, 25–27], a similar phenomenon was observed, where XNA chimeras with the lowest T_m_ were better able to silence gene expression compared to all-FANA sequences. While the trends are the same, the mechanisms underlying such improvements are fundamentally different. For example, in reference [18], the effectiveness of the X-ASOs was determined by measuring the reduction in bioluminescence, corresponding to reduced cellular gene expression. However, in our study, the sequence-specific XNAzyme-mediated cleavage of *in vitro* transcribed lsRNA was measured via dPAGE, free from any cellular components like RNases or other RNA-binding proteins. Thus, the unique LNA–FRNA–FANA pattern of XdZ-1 and -2 provides a distinct and unexpected advantage to lsRNA accessibility.

### RNA-cleavage activity comparison of XdZ-2 to other lsRNA targets and accessibility methods

After identifying XdZ-2 (possessing the LNA–FRNA–FANA arm pattern of XNA C2) as the best XNAzyme out of the 5 XdZ constructs tested with the 831-nt *ORF3a* lsRNA target, we investigated its generalizability to other lsRNAs and comparative performance to other methods of improving accessibility to lsRNA. We first designed X10-23B and XdZ-2 constructs that targeted structured sites within two different lsRNA transcripts from SARS-CoV-2. The lsRNA transcripts, target sites, and 10–23 DNAzymes were identical to those used in a previous study that used ASOs to improve lsRNA accessibility to 10–23 DNAzymes [21]. The first set of XNAzymes (X10-23S and XdZ-2S) targeted a 566-nt lsRNA transcript from the gene encoding for the Spike protein and the second set (X10-23N and XdZ-2N) targeted a 584-nt lsRNA transcript from the *NSP8* gene. For each system, including the original 831-nt lsRNA transcript from *ORF3a*, the same LNA–FRNA–FANA arm pattern of XdZ-2 was used so that a direct comparison in lsRNA cleavage activity could be made. We also compared the lsRNA cleavage activity between each XdZ-2 and X10-23 construct, as well as the corresponding 10–23 DNAzyme with and without ASOs [21].

For the original 831-nt *ORF3a* lsRNA transcript, the unmodified, all-DNA [16 + 8] 10–23 DNAzyme cleaved the lsRNA target site with a *k*_obs_ of 0.00016 min^−1^ (Supplementary Fig. S5). The addition of ASOs to the lsRNA, upstream and downstream of the DNAzyme target site, improved activity by 106-fold (*k*_obs_ = 0.017 min^−1^) as reported in the study [21]. Including the lsRNA cleavage activities of X10-23B (*k*_obs_ = 0.00028 min^−1^) (Fig. 7A) and XdZ-2 (*k*_obs_ = 0.023 min^−1^) (Fig. 7B), the order of lsRNA-cleavage activity by each enzyme for this system follows 10–23 << X10-23 < 10–23 + ASOs < XdZ-2, showing that the XNA chimera substrate recognition arms of XdZ-2 are the best strategy to improve 10–23 access to lsRNA compared to X10-23 or [16 + 8] all-DNA 10–23 with ASOs.

With 566-nt *Spike* lsRNA, the unmodified all-DNA [16 + 8] 10–23 cleaved the lsRNA with a *k*_obs_ of 0.000034 min^−1^ and when an ASO was added (only the downstream ASO was required for this system), the rate of lsRNA cleavage improved 256-fold (*k*_obs_ of 0.0087 min^−1^) [21] (Supplementary Fig. S5). Contrary to the system targeting the 831-nt *ORF3a* lsRNA, in the 566-nt *Spike* lsRNA system, the all-DNA [16 + 8] 10–23 had a higher rate of lsRNA cleavage with the ASO compared to X10-23S or XdZ-2S, with a *k*_obs_ of 0.0025 min^−1^ (Fig. 7C) and 0.0047 min^−1^ (Fig. 7D), respectively. Although XdZ-2 possessed 1.8-fold higher 566-nt *Spike* lsRNA cleavage activity compared to X10-23S, for this system, the order of lsRNA-cleavage activity by each enzyme follows 10–23 << X10-23S < XdZ-2S < 10–23 + ASO, indicating that the ASO strategy was more effective than XNAs at improving 10–23 access to lsRNA, although the rates of the latter three are relatively close.

By contrast, for the 584-nt *NSP8* lsRNA system, the rate of lsRNA-cleavage by the unmodified all-DNA [16 + 8] 10–23 with ASOs (*k*_obs_ = 0.078 min^−1^) was significantly higher than unmodified 10–23 (*k*_obs_ = 0.000039 min^−1^) (Supplementary Fig. S5), X10-23N (*k*_obs_ = 0.00043 min^−1^) (Fig. 7E), and XdZ-2N (*k*_obs_ = 0.00043 min^−1^) (Fig. 7F), with an overall activity order of 10–23 < X10-23N < XdZ-2N << 10–23 + ASOs. Despite possessing the same rate of lsRNA cleavage according to curve fitting to the one-phase exponential association equation (using GraphPad), XdZ-2N cleaved 58% of lsRNA after 48 h (Y_48_ = 58%) compared to only 22% cleavage (Y_48_ = 22%) by X10-23N; however, a 48 h incubation is likely unrealistic for most use cases. Compared to the unmodified all-DNA version, both XdZ-2N and X10-23N cleaved the 584-nt *NSP8* lsRNA 10-fold faster. However, like the 566-nt *Spike* lsRNA system, 10–23N with ASOs offered the best rate enhancement (*k*_obs_ = 0.078 min^−1^), with 2000-fold improvement over the same reaction without ASOs and 181-fold enhancement over the activity of both XdZ-2N and X10-23N.

In terms of generalizability, all three XdZ-2 variants cleaved their respective lsRNA targets faster than their [8 + 8] X10-23 and [16 + 8] all-DNA 10–23 counterparts, albeit with significant variation. While XdZ-2 was the best at cleaving 831-nt *ORF3a* lsRNA, the addition of ASOs to the all-DNA [16 + 8] 10–23 was more effective than XdZ-2 for 566-nt *Spike* lsRNA and especially for 584-nt *NSP8* lsRNA (a summary of each strategy is shown in Supplementary Table S2). For practical use cases, such as improving access to viral RNA targets in clinical samples for diagnostics, a trade-off analysis between each strategy should be considered. For example, while ASOs may offer improved access to lsRNA compared to XdZ-2, overall costs may be higher (2–3 oligonucleotides with ASOs instead of 1 with XdZ-2). Moreover, for *in vivo* therapeutics and in minimally processed clinical samples, the ASO all-DNA systems will likely be more susceptible to degradation by nucleases, limiting their effectiveness.

### Comparing X10-23 and XdZ-2 enzymes under quasi-physiological and diagnostic reaction conditions

Magnesium concentration and reaction temperature have significant impacts on the activity of the 10–23 DNAzyme, participating in both catalytic and structural processes [48, 49]. Higher temperatures and Mg^2+^ concentrations typically correlate with higher RNA-cleavage activity [2]. Kinetic experiments from the previous sections were conducted in buffer containing relatively high amounts of Mg^2+^ (10 mM), but under physiological conditions, Mg^2+^ levels exist around 0.5–2 mM [50], reducing the effectiveness of the 10–23 DNAzyme for *in vivo* therapeutic applications [51]. Recent studies have shown that FANAzymes, such as the X10-23 enzyme, can better retain RNA-cleavage activity at 1 mM Mg^2+^ and 37°C [4, 5]. For *in vitro* diagnostic applications, the reaction conditions are typically reversed, with Mg^2+^ levels artificially increased to as high as 25 mM, greatly improving 10–23 activity, but with lower incubation temperatures (23°C) to be more compatible with point-of-care diagnostics. As a result, we tested the lsRNA-cleavage activity of XdZ-2 under both quasi-physiological and ideal diagnostic reaction conditions, comparing it to X10-23B.

**Figure 8.**
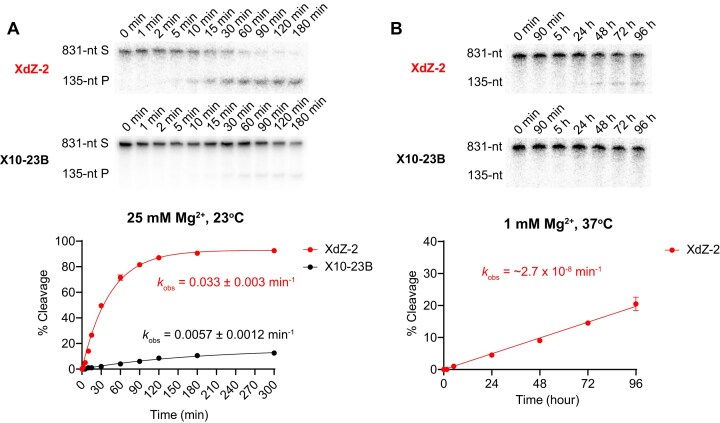
RNA cleavage activity comparison of X10-23B and XdZ-2 under typical diagnostic reaction conditions of 25 mM Mg^2+^ at 23°C (**A**) and quasi-physiological conditions of 1 mM Mg^2+^ at 37°C (**B**). Each XNAzyme was tested against an 831-nt lsRNA substrate from the *ORF3a* gene of SARS-CoV-2. Reactions were resolved in 10% dPAGE with 8 M urea.

We first performed kinetic experiments with X10-23B and XdZ-2 using the same 831-nt *ORF3a* lsRNA substrate under diagnostic conditions of 25 mM Mg^2+^ and 23°C. Comparing *k*_obs_ values, XdZ-2 was nearly six-fold faster than X10-23B (*k*_obs_ = 0.033 min^−1^ vs *k*_obs_ = 0.0057 min^−1^), showing that XdZ-2 is a more suitable catalyst compared to enzyme X10-23 at accessing lsRNA for diagnostic applications (Fig. 8A). Compared to the original kinetic reaction performed at 10 mM Mg^2+^ and 23°C (Fig. 7B and Supplementary Fig. S3), XdZ-2 showed 1.4-fold higher lsRNA-cleavage activity (*k*_obs_ = 0.033 min^−1^ vs *k*_obs_ = 0.023 min^−1^), indicating that 25 mM Mg^2+^ provided only moderate improvements to lsRNA-cleavage activity by XdZ-2. We also tested Mg^2+^ concentrations of 5 mM and 2 mM at 23°C to identify the lower limits of Mg^2+^ concentrations at 23°C for diagnostic applications and found a slight reduction in lsRNA-cleavage activity (*k*_obs_ = 0.013 min^−1^) at 5 mM, with a significant reduction (*k*_obs_ = 0.00062 min^−1^) at 2 mM (Supplementary Fig. S6).

Next, we performed kinetics with X10-23B and XdZ-2 for the same 831-nt *ORF3a* lsRNA substrate under quasi-physiological conditions of 1 mM Mg^2+^ and 37°C. Compared to the lsRNA-cleavage activity under diagnostic conditions, the activity of XdZ-2 was significantly reduced, cleaving the lsRNA target with a *k*_obs_ of ~2.8 × 10^−8^ min^−1^ (Fig. 8B). While XdZ-2 provided a 1000-fold increase in the rate lsRNA cleavage compared to the rate of uncatalyzed RNA transesterification [52] and was more active than X10-23B, the rate of lsRNA cleavage was still over 150 000-fold slower than the *in vivo* rate of decay for certain lsRNAs, such as *KRAS* mRNA, which turns over at a rate of 0.0042 min^−1^ [53]. Moreover, XdZ-2 was over 400 000-fold slower compared to existing FANAzymes such as FR6_1, which achieved a *k*_obs_ of 0.012 min^−1^ with a ∼2100-nt *KRAS* mRNA target under the same reaction conditions (Supplementary Table S3) [5].

In contrast to XdZ-2 activity at high [Mg^2+^], additional optimization will be required to maximize activity under low [Mg^2+^] quasi-physiological conditions. Increasing the substrate recognition arm length of XdZ-2 is likely the simplest and most effective strategy, given that FR6_1 possessed [10 + 10] FANA substrate recognition arms, ∼23% larger than the 31-mer XdZ-2 with a substrate recognition arm length of [8 + 8]. The [10 + 10] variant of XdZ-2 may improve the lsRNA-cleavage activity relative to FR6_1 under physiological conditions, but a balance between cost of synthesis and activity must ultimately be considered for each application.

Expanding beyond RNA-cleaving DNAzymes or XNAzymes such as 10–23 or FR6_1, we hypothesize that any nucleic acid-based catalyst with issues accessing large, structured RNA targets may benefit from the substrate recognition arm design of XdZ-2, so long as it possesses modifiable arms that function in the *trans* configuration. An example includes previously mentioned FR6_1, which may have improved lsRNA cleavage activity with the XNA C2 arm sequence [5]. For RNA-ligating FANAzymes such as FpImR4_2, which ligates two separate RNA substrates, XNA C2 arm substitution may expand the repertoire of RNA substrates to include larger, more structured targets [13]. For DNAzymes or XNAzymes without substrate recognition arms or strict RNA substrate sequence requirements, XNA substitution could improve catalytic activity but may be inhibitory if the substrate-binding region is too short (low *k*_on_) or too long (low *k*_off_). For DNAzymes or XNAzymes that target DNA or XNA substrates, we do not expect much improvement in catalytic activity with XNA C2 arms, as DNA and XNA do not possess structural elements as extensive as those of RNA.

Overall, we showed that recently reported XNAzymes, such as X10-23, are significantly less effective when targeting structured target sites within lsRNA of biological origin. To improve accessibility and generalizability of X10-23 to lsRNA targets, while also considering synthesis cost and substrate recognition arm length, we tested several X10-23 variants with chimeric XNA substrate recognition arms possessing high RNA binding strength. We identified an XNAzyme, XdZ-2, with a distinct arm pattern of LNA–FRNA–FANA that facilitated greater activity and access to an lsRNA target compared to the all-FANA substrate recognition arms of X10-23. Although more active than X10-23 across all lsRNA targets, XdZ-2 was less active compared to the previously reported all-DNA 10–23 + ASO strategy for 2 of 3 systems. In these cases, it is possible that substrate recognition arms could be increased to improve hybridization, or other XNA patterns (XNA C1, C3-5) could be more effective at gaining access to their respective lsRNA targets and should be explored if the XNA C2 pattern is ineffective. However, its relatively short [8 + 8] substrate recognition arm length provides benefits of lower synthesis cost, higher stability, and more efficient product release for downstream reactions. Moreover, its ability to cleave lsRNA under high (25 mM) and low (1 mM) Mg^2+^ levels more effectively than X10-23 highlights the utility of XdZ-2 for future applications.

## Supplementary Material

gkaf1449_Supplemental_File

## Data Availability

The data underlying this article are available in the article and in its online supplementary data.
