# Supplementary material for: A modified X10-23 DNAzyme that can better access large, structured RNA targets
Source: Nucleic Acids Res. 2026 Jan 6;54(1):gkaf1449. doi: 10.1093/nar/gkaf1449 (PMC12774633; doi:10.1093/nar/gkaf1449)
Supplement: gkaf1449_Supplemental_File [file gkaf1449_supplemental_file.pdf]

A modified X10-23 DNAzyme that can better access large, structured RNA targets

Supplementary Information

Connor Nurmi[12], Halle M. Barber[3], Harneesh Kaur[3], John D. Brennan,[2] Masad J. Damha[3,*], Yingfu Li[1,*]

* To whom correspondence should be addressed. Email: liying@mcmaster.ca, masad.damha@mcgill.ca

1. Department of Biochemistry and Biomedical Sciences, McMaster University, Ontario L8S 4L8, Canada
2. Biointerfaces Institute, McMaster University, Ontario L8S 4L8, Canada
3. Department of Chemistry, McGill University, Montreal, Quebec H3A 0G4, Canada

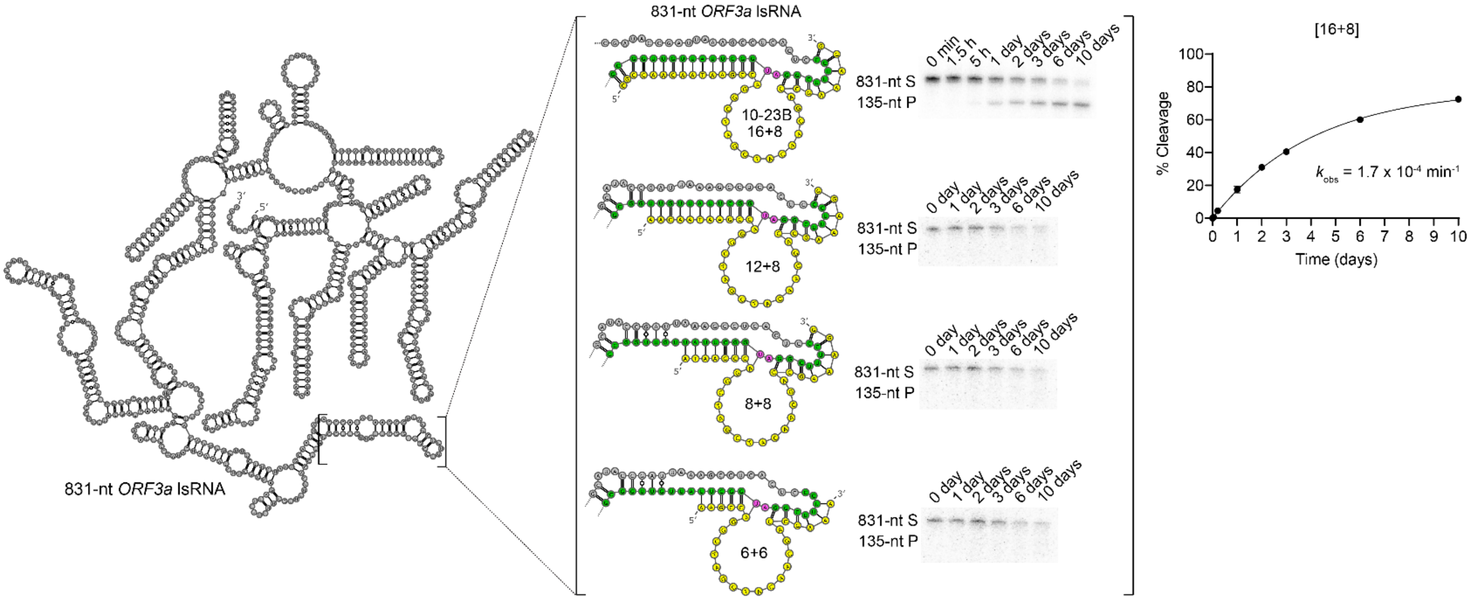

Figure S1. DNAzyme kinetics of 10-23B with substrate recognition arm lengths of [6+6], [8+8], [12+8] and [16+8] for 831-nt lsRNA from the *ORF3a* gene of SARS-CoV-2. Reactions were performed at room temperature in buffer containing 10 mM Mg^{2+} and were separated by 10% denaturing PAGE with 8 M urea. Only the [16+8] 10-23B construct showed activity after 10 days.

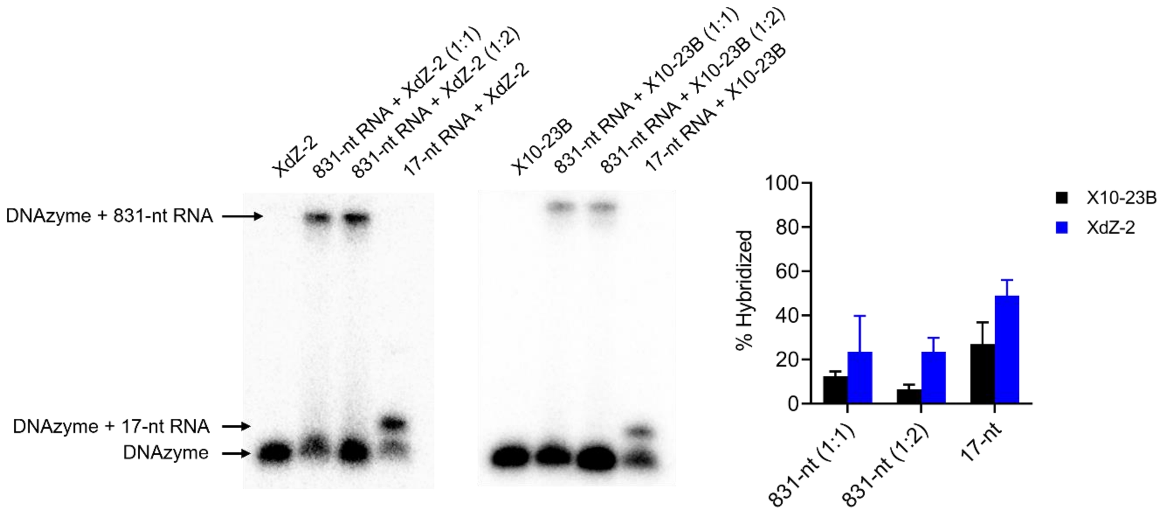

Figure S2. Electrophoretic mobility shift assay (EMSA) for XdZ-2 and X10-23 with short 17-nt RNA and 831-nt lsRNA. DNAzyme constructs and RNA substrates were heated at 90 °C for 1 min then cooled at room temperature for 10 min to allow folding to occur. Following the addition of 1 × buffer 1 on ice, reactions were incubated at 4°C for 5 days. Reactions were then separated by 10% native PAGE.

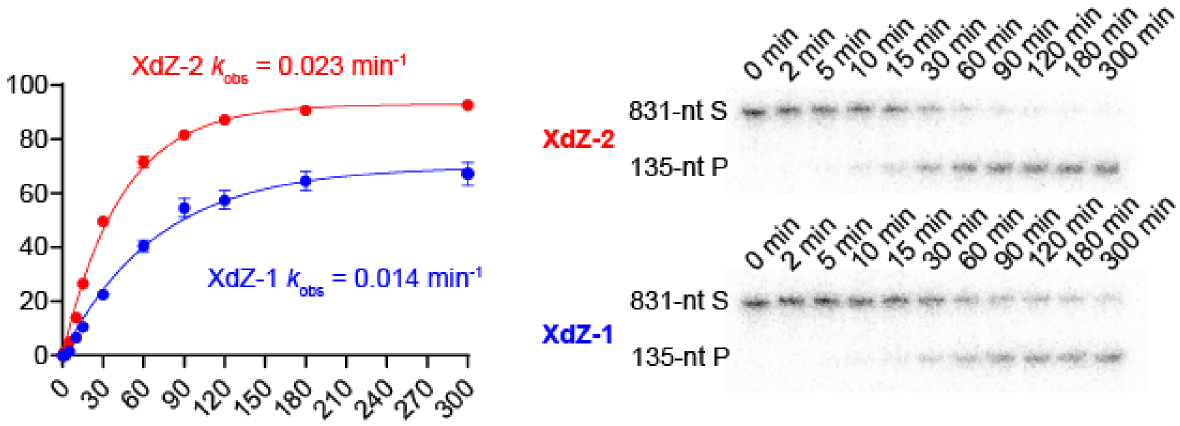

Figure S3. XNAzyme kinetics of XdZ-1 and XdZ-2 for 831-nt lsRNA from the *ORF3a* gene of SARS-CoV-2. Reactions were performed at room temperature in buffer containing 10 mM Mg^{2+} and were separated by 10% denaturing PAGE with 8 M urea.

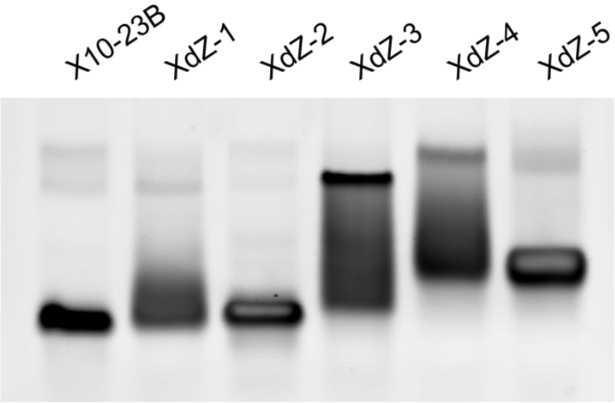

Figure S4. Native folding of the 5 XdZ chimeras (XdZ-1 to 5) and X10-23B with substrate recognition arm length of [8+8]. XNAzyme constructs were heated at 90 °C for 1 min then cooled at room temperature for 10 min to allow folding to occur. XNAzyme constructs were separated by 15% native PAGE.

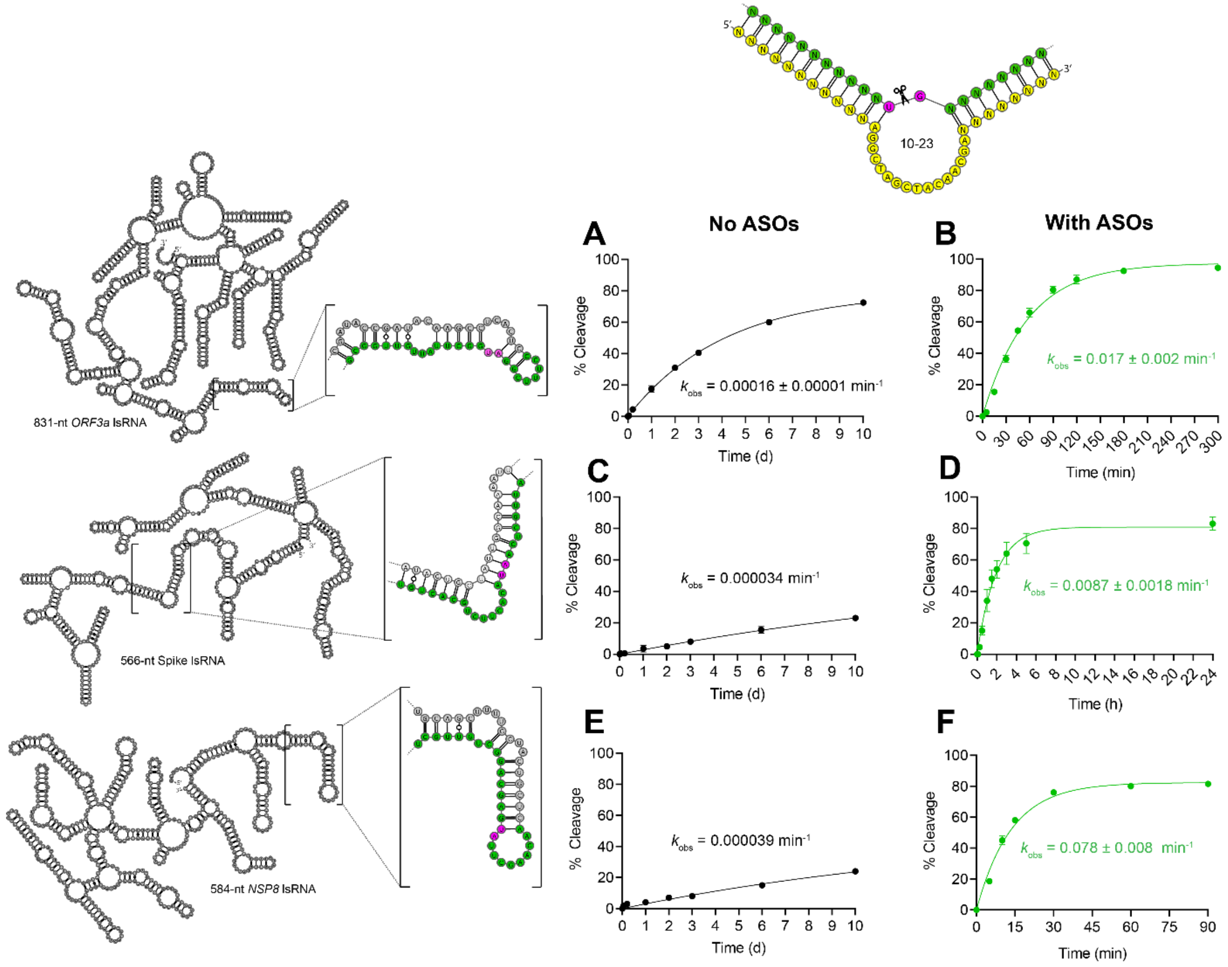

Figure S5. Antisense DNA oligonucleotide (ASO)-mediated improvement of 10-23 DNAzyme accessibility to lsRNA targets from various genes of SARS-CoV-2 based on reference (1). Three different 10-23 DNAzymes targeted three separate lsRNA transcripts, including an 831-nt lsRNA transcript from the *ORF3a* gene (**A & B**), a 566-nt lsRNA transcript from the *Spike* gene (**C & D**) and a 584-nt lsRNA transcript from the *NSP8* gene (**E & F**). DNAzyme kinetic reactions with the *ORF3a* and *NSP8* lsRNA transcripts employed two ASOs located upstream and downstream of the DNAzyme target site, while the *Spike* lsRNA used only one located downstream of the DNAzyme target site. All reactions were conducted at 23°C in buffer containing 10 mM Mg^{2+}.

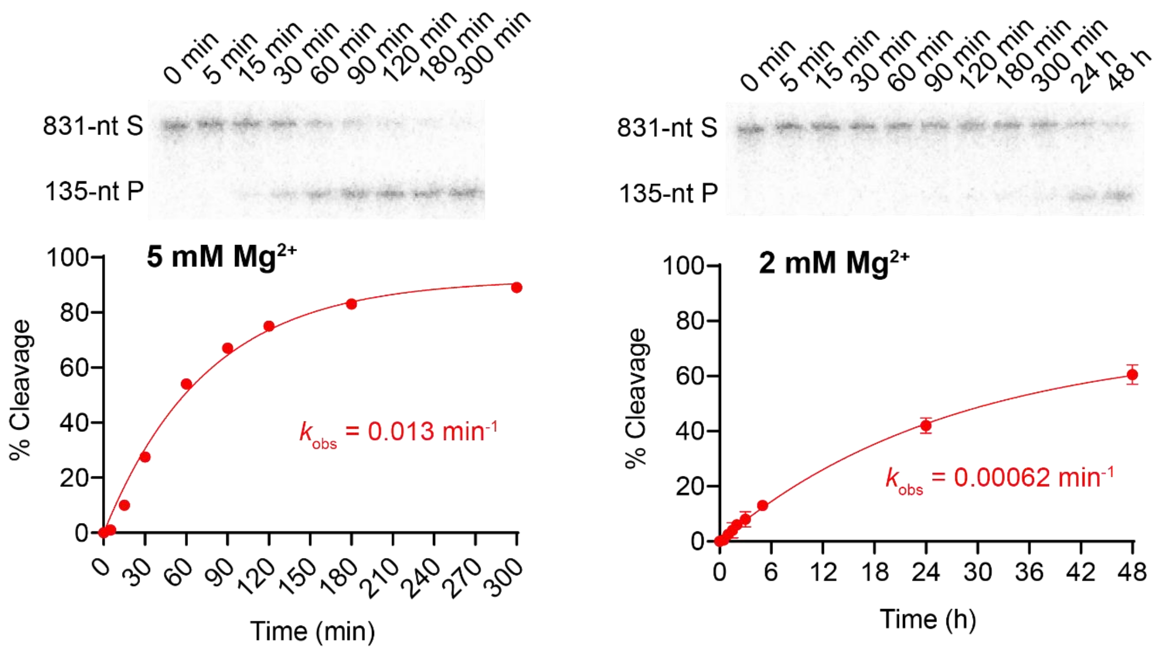

Figure S6. Magnesium titration of XdZ-2 with 831-nt *ORF3a* lsRNA. XdZ-2 kinetic reactions were conducted at room temperature in duplicate and analyzed by 10% PAGE with 8 M urea.

Table S1. List of RNA substrate sequences used in this study.

Oligonucleotide	Sequence 5′→3′	Length
RNA substrate for T_m and CD studies	CCUUUCGGUGGCUUAU	16
X10-23B 17-nt *ORF3a* RNA Substrate for T_m and CD studies	CCUUUCGGAdTGGCUUAU	17
X10-23A 13-nt *ORF3a* RNA Substrate	AAACCCAUUACUU	13
X10-23B 13-nt *ORF3a* RNA Substrate	UUUCGGAUGGCUU	13
X10-23B 17-nt *ORF3a* RNA Substrate	CCUUUCGGAUGGCUUAU	17
831-nt *ORF3a* lsRNA Substrate from SARS-CoV-2 25393/26220	GGGAUGGAUUUGUUUAUGAGAAUCUUCACAAUUGGAACUGUAA<br>CUUUGAAGCAAGGUGAAAUCAAGGAUGCUACUCCUUCAGAUUU<br>UGUUCGCGCUACUGCAACGAUACCGAUACAAGCCUCACUCCCU<br>UUCGGAUGGCUUAUUGUUGGCGUUGCACUUCUUGCUGUUUUU<br>CAGAGCGCUUCCAAAAUCAUAACCCUCAAAAAGAGAUGGCAACU<br>AGCACUCUCCAAGGGUGUUCACUUUGUUUGCAACUUGCUGUUG<br>UUGUUUGUAACAGUUUACUCACACCUUUUGCUCGUUGCUGCUG<br>GCCUUGAAGCCCCUUUUCUCUAUCUUUAUGCUUUAGUCUACUU<br>CUUGCAGAGUAUAAACUUUGUAAGAAUAAUAAUGAGGCUUUGG<br>CUUUGCUGGAAAUGCCGUUCCAAAAACCCAUUACUUUAUGAUG<br>CCAACUAUUUUCUUUGCUGGCAUACUAAUUGUUACGACUAUUG<br>UAUACCUUACAAUAGUGUAACUUCUUCAAUUGUCAUUACUUCA<br>GGUGAUGGCACAACAAGUCCUAUUUCUGAACAUGACUACCAGA<br>UUGGUGGUUAUACUGAAAAAUGGGAAUCUGGAGUAAAAGACUG<br>UGUUGUAUUACACAGUUACUUCACUUCAGACUAUUACCAGCUG<br>UACUCAACUCAAUUGAGUACAGACACUGGUGUUGAACAUGUUA<br>CCUUCUUCAUCUACAAUAAAAUUGUUGAUGAGCCUGAAGAACA<br>UGUCCAAAUUCACACAAUCGACGGUUCAUCCGGAGUUGUUAAU<br>CCAGUAAUGGAACCAAUUUAUGAUGAACCGACGACGACUACUA<br>GCGUGCCUUUGUAA	831
566-nt *Spike* lsRNA Substrate from SARS-CoV-2 24108/24665	GGGUUUCCCCAUUUGUGCACAAAAGUUUAACGGCCUUACUGUU<br>UUGCCACCUUUGCUCACAGAUGAAAUGAUUGCUCAAUACACUU<br>CUGCACUGUUAGCGGGUACAAUCACUUCUGGUUGGACCUUUG<br>GUGCAGGUGCUGCAUUACAAAUACCAUUUGCUAUGCAAAUGGC<br>UUAUAGGUUUAAUGGUAUUGGAGUUACACAGAAUGUUCUCUAU<br>GAGAACCAAAAAUUGAUUGCCAACCAAUUUAAUAGUGCUAUUG<br>GCAAAAUUCAAGACUCACUUUCUUCCACAGCAAGUGCACUUGG<br>AAAACUUCAAGAUGUGGUCAACCAAAAUGCACAAGCUUUAAACA<br>CGCUUGUUAAACAACUUAGCUCCAAUUUUGGUGCAAUUUCAAG<br>UGUUUUAAAUGAUAUCCUUUCACGUCUUGACAAAGUUGAGGCU<br>GAAGUGCAAAUUGAUAGGUUGATCACAGGCAGACUUCAAAGUU<br>UGCAGACAUAUGUGACUCAACAAUUAAUUAGAGCUGCAGAAAU	566

	CAGAGCUUCUGCUAAUCUUGCUGCUACUAAAAUGUCAGAGUGU GUACUUG	
584-nt *NSP8* IsRNA Substrate from SARS-CoV-2 12098/12679	GGGCCUCAGAGUUUAGUUCCCUUCCAUCAUAUGCAGCUUUUGC UACUGCUCAAGAAGCUUAUGAGCAGGCUGUUGCUAAUGGUGAU UCUGAAGUUGUUCUUAAAAAGUUGAAGAAGUCUUUGAAUGUGG CUAAAUCUGAAUUUGACCGUGAUGCAGCCAUGCAACGUAAGUU GGAAAAGAUGGCUGAUCAAGCUAUGACCCAAAUGUAUAAACAG GCUAGAUCUGAGGACAAGAGGGCAAAAGUUACUAGUGCUAUGC AGACAAUGCUUUUCACUAUGCUUAGAAAGUUGGAUAAUGAUGC ACUCAACAACAUUAUCAACAAUGCAAGAGAUGGUUGUGUUCCC UUGAACAUAAUACCUCUUACAACAGCAGCCAAACUAAUGGUUGU CAUACCAGACUAUAACACAUAUAAAAAUACGUGUGAUGGUACAA CAUUUACUUAUGCAUCAGCAUUGUGGGAAAUCCAACAGGUUGU AGAUGCAGAUAGUAAAAUUGUUCAACUUAGUGAAAUUAGUAUG GACAAUUCACCUAAUUUAGCAUGGCCUCUUAUUGUAACAGCUU UAAGGGCCAAUUCUGCUGUCAAA	584

Table S2: Summary of the observed rate constant (k_{obs}, min^{-1}) of each DNAzyme or XNAzyme construct from the three lsRNA transcripts from various genes of SARS-CoV-2. Data from the all-DNA 10-23 and all-DNA 10-23 + ASOs is from reference (1).

	831-nt *ORF3a* lsRNA	584-nt *NSP8* lsRNA	566-nt *Spike* lsRNA
All-DNA 10-23	1.6×10^{-4}	3.9×10^{-5}	6.3×10^{-5}
XdZ-2	**2.3×10^{-2}**	4.3×10^{-4}	4.7×10^{-3}
X10-23	2.8×10^{-4}	4.3×10^{-4}	2.5×10^{-3}
All-DNA 10-23 + ASOs	1.7×10^{-2}	**7.8×10^{-2}**	**8.7×10^{-3}**

Table S3: Other reported XNAzymes that targeted large, structured RNA substrates with reported kinetic parameters. DV28 and Lz9-745 possessed only XNA substrate recognition arms with a full-DNA 10-23 catalytic core.

**XNAzyme**	**RNA substrate length (nt)**	**Substrate recognition arm length**	**Substrate recognition arm XNA**	**Diagnostic ([Mg^{2+}] = 25 mM, 23°C) k_{obs} (min^{-1})**	**Diagnostic ([Mg^{2+}] = 25 mM, 23°C) Y_{60} (min)**	**Therapeutic ([Mg^{2+}] = 1 mM, 37°C) k_{obs} (min^{-1})**	**Therapeutic ([Mg^{2+}] = 1 mM, 37°C) Y_{60} (min)**	**Ref**
XdZ-2	831	8+8	LNA, FRNA, FANA	0.033	69%	2.7×10^{-8}	0%	This study
DV28 (10-23)	~2600	7+5	2′-OMe	-	~42%	-	-	(2)
Lz9-745 (10-23)	2904	8+8	LNA	-	13%	-	-	(3)
FR6_1	2100	10+10	FANA	0.08*	~24%	0.012	~22%	(4)

References

1. Nurmi,C., Gu,J., Mathai,A., Brennan,J.D. and Li,Y. (2024) Making target sites in large structured RNAs accessible to RNA-cleaving DNAzymes through hybridization with synthetic DNA oligonucleotides. *Nucleic Acids Research*, **52**, 11177–11187.

2. Schubert,S., Fürste,J.P., Werk,D., Grunert,H.-P., Zeichhardt,H., Erdmann,V.A. and Kurreck,J. (2004) Gaining Target Access for Deoxyribozymes. *Journal of Molecular Biology*, **339**, 355–363.

3. Vester,B., Hansen,L.H., Bo Lundberg,L., Babu,B.R., Sørensen,M.D., Wengel,J. and Douthwaite,S. (2006) Locked nucleoside analogues expand the potential of DNAzymes to cleave structured RNA targets. *BMC Mol Biol*, **7**, 19.

4. Taylor,A.I., Wan,C.J.K., Donde,M.J., Peak-Chew,S.-Y. and Holliger,P. (2022) A modular XNAzyme cleaves long, structured RNAs under physiological conditions and enables allele-specific gene silencing. *Nat. Chem.*, **14**, 1295–1305.
